# Editorial: Plant responses to bacterial quorum sensing molecules

**DOI:** 10.3389/fpls.2015.00643

**Published:** 2015-08-19

**Authors:** Anton Hartmann, Adam Schikora

**Affiliations:** ^1^Research Unit Microbe-Plant Interactions, Department of Environmental Sciences, Helmholtz Zentrum München - German Research Center for Environmental Health GmbHNeuherberg, Germany; ^2^Research Centre for Biosystems, Land Use and Nutrition (IFZ), Institute for Phytopathology, Justus Liebig University GiessenGiessen, Germany

**Keywords:** quorum sensing, AHL-priming, PGPR-bacteria, LuxR solos, inter-kingdom communication

Since the discovery of the phenomenon in the 70s and the introduction of the term two decades later (Nealson et al., 1970; Fuqua and Winans, 1994), our knowledge about quorum sensing (QS) in bacterial populations has reached a quite high level. Moreover, throughout the last years progressively more information was available on the role of QS molecules during the inter-kingdom interactions between bacteria and the eukaryotic hosts, like humans, animals, or plants. Much of this knowledge was collected in the medical field, where QS molecules play an essential role in the pathogenicity of certain bacteria toward humans. However, also plant-bacteria interactions are influenced by the presence of QS molecules and we commence to understand the underlying mechanisms. Most knowledge about the effects of *N*-acyl-homoserine lactones (AHL), the major group of QS-signaling molecules in Gram-negative bacteria, on plants, has been gathered for the model plant *Arabidopsis thaliana*. In this plant, one has to discriminate between the perception and effects of short carbon-chain (C4-, C6-, C8-) and long carbon-chain (C12-, and C14-) AHLs (Schenk et al., 2012; Hartmann et al., 2014). While the short carbon-chain AHLs are perceived by the G-protein-coupled receptor (GPCR) in *Arabidopsis* (Liu et al., 2012), the receptor mechanism of the lipophilic long carbon-chain AHLs is yet unknown. Interestingly, the long carbon-chain AHLs, e.g., the 3-oxo-C14-HSL, prime plants for enhanced resistance, a phenomenon based on oxylipin and salicylic acid dependent signaling pathway and called AHL-priming (Schenk and Schikora, 2015).

In this research topic, authors present reviews and original research papers in different areas related to the role of QS molecules during the interaction between bacteria and plants. These contributions can be clustered in three different categories: specific perception and cellular signaling, ecological effects, and impact of QS molecules on the host physiology.

Today, one of the best-studied systems in the field of interaction between bacterial QS molecules and eukaryotes is the interaction between the 3-oxo-C12-HSL, an AHL produced by the human pathogen *Pseudomonas aeruginosa*, and humans. In their review, Holm and Vikström present the newest advances in this field. Moreover, based on own research, the authors propose that the IQ-motif-containing GTPase-activating protein IQGAP1 interacts with the 3-oxo-C12-HSL and therefore could be a eukaryotic target for bacterial AHLs (Holm and Vikström, 2014). In contrast, the bacterial perception mechanism of AHLs is well understood. The perception is based on LuxR-type receptors. Additionally, LuxR solos, i.e., LuxR receptors that exist in the absence of the usual LuxI counter partner, which is responsible for the synthesis of the actual AHL molecule, evolved (or were acquired) in order to allow perception of QS molecules in bacteria that do not produce AHLs. LuxR solos have acquired also further functions, like binding different exogenous and endogenous signals. A special class of LuxR solos can perceive QS molecules other than AHLs and even plant-originated molecules (Patel et al., 2013). In addition to the perception of plant-originated molecules via the QS receptors in bacteria, plants can perceive AHLs in a very specific manner. Veliz-Vallejos and colleagues describe the impact of 3-oxo-C14-HSL, an AHL produced by *Sinorhizobium meliloti*, on the nodulation of the potential host plant *Medicago truncatula* (Veliz-Vallejos et al., 2014). Very striking was the fact that the increased numbers of nodules were observed only upon treatment with 3-oxo-C14-HLS, other AHLs (produced by other bacteria) or the use of a different leguminous plant resulted in no effect. These results point to a very precise perception system in legumes. A central role of calcium signaling and calmodulin in the response of *Arabidopsis* to short carbon-chain AHLs is shown in the report by Zhao et al. (2015). The authors describe that calmodulin antagonists abolished the beneficial effect of 3-oxo-C6-HSL on root elongation and, at the same time, that the level of CaM increased after 3-oxo-C6-HSL treatment. Moreover, mutants in *AtCaM* genes did not respond to the treatment. This strongly indicates that calmodulin plays a central role in the perception of short carbon-chained AHLs.

On a different scale, AHL treatment seems to have impact on the host metabolism and even reproduction systems. A study on C6-, C8-, and C10-AHLs conducted on barley (*Hordeum vulgare*) and the tropical leguminous crop yam bean (*Pachyrhizus erosus*) revealed that these AHLs influence the activity of specific detoxification enzymes, namely glutathione S-transferase and dehydroascorbate reductase in barley, however much less in yam bean (Götz-Rösch et al., 2015). This difference might be due to reduced transport of AHLs to the shoot in yam bean caused by a lactonase-dependent degradation. In addition, short-chained C4- and C6-AHLs produced by bacteria in biofilms on green macroalgae *Ulva* and red macroalgae *Gracilaria* stimulated the release of carpospores (Singh et al., 2015). Interestingly, algae's cystocarps and the cystocarp-bearing plantlets treated with AHLs had a distinct protein pattern with several, yet unidentified, proteins induced specifically after AHL-treatment.

This collection of articles highlights the complex interactions between bacteria, plants, and animals, and reflects the different levels at which QS molecules are involved in these interactions. It is becoming clear that the plant-associated microbiomes will play a central role in the development of new strategies aiming at the improvement of our food supply and food quality. To understand the QS and the impact of QS molecules on the outcome of bacteria-plant interactions will be a challenge for the coming years. Moreover, the new insights on inter-kingdom communication gathered during these studies will be relevant for an advanced understanding of QS in pathogenicity of bacterial human pathogens.

## Conflict of interest statement

The authors declare that the research was conducted in the absence of any commercial or financial relationships that could be construed as a potential conflict of interest.
